# The Colon of the Horse—Its Diseases and Derangements

**Published:** 1887-10

**Authors:** Thomas Walley

**Affiliations:** Principal Royal (Dick) Veterinary College, Edinburgh


### Art. XXXI.—THE COLON OF THE HORSE—ITS DISEASES AND DERANGEMENTS.

BY THOMAS WALLEY, M. R. C. V. S.

*Principal Royal (Dick) Veterinary College, Edinburgh.*

At the first blush it might be thought that this gut offers for consideration only a very limited amount of material, but I venture to say that there is no organ, nor any part of a single organ in the body of the horse that is more frequently the seat of functional or organic derangement. That it should be liable to mechanical displacement is not a matter for wonder when its great volume and capacity, the bulkiness of its contents and its anatomical peculiarities are taken into consideration. As a matter of wisdom, one would scarcely have given to an animal like the horse, such a voluminous organ as this, and the only apology that can be offered for such an arrangement is that it may serve the purpose of retaining the vegetable matter ingested for a sufficient length of time to ensure the assimilation of its nutrient elements; but allowing this, nature, according to our limited ken, was still in fault, inasmuch as she did not take steps for the more effectual anchoring of such a voluminous organ in a cavity like the abdomen, the capacity of which is ever varying. The wonder to me is, not that the colon is so frequently displaced, but rather that is so seldom displaced; for very little reflection seems to show, I think, that a mere stumble, an awkward position of the body or the assumption of the dorsal posture would be amply sufficient to throw it out of its normal situation, and particularly so when its contents are of a solid or semi-fluid character. That every lesion of this organ can be distinguished during life, no veterinary practitioner will assert, but that we possess tolerably reliable diagnostic signs of some of the lesions in which it is engaged, I shall, I think, be able conclusively to show, while I hope to dispel one or two common fallacies which exist in connection with this organ.

In treating of colic disorders it must be borne in mind that I refer mainly to the double colon; incidentally, the single colon, and the cæcum will receive a small share of attention also. I shall deal with the subject in much the same manner as that adopted in treating of Œsophagal diseases, and as in the case of that organ, so here, I shall first consider functional derangements and symptomatic conditions.

Under normal conditions a constant movement of a determinate character (*peristalsis*) goes on in the colon, whether a reversal of this movement—*anti-peristalsis*—ever occurs may be a mooted point; that such may take place, judging from what we know of experimental enquiry, is possible, particularly under the influence of direct stimulants, *e. g.*, the sodium salts. The normal movement of the bowels cannot be taken cognizance of either visually or by manual examination, but inasmuch as it is accompanied by a more or less rapid displacement of the contents of the colon, certain sounds are produced which are termed *borborygmi* (G rolling and tumbling) and their nature is determined by the character of the colic contents, *i. e.*, if comparatively solid we have a rolling or dull sound produced, if fluid or semi-fluid, a rattling or gurgling sound ; if gas, a rumbling sound, while in colliquative diarrhœa, where the solid matter has been evacuated, a clear metallic or tinkling sound is heard. Borborygmus is by many accepted as a measure of the activity of the bowels, and is often eagerly anticipated in cases of colic impaction as a sign that the bowel is about to react, but it is not in every instance reliable to this end, especially where gas is present, as this may produce a rumbling sound by its own independent movements. Moreover, when the posterior bowels are absolutely fixed, as in cases of twist, borborygmus is frequently heard in the uninvolved parts, and even in general intestinal paralysis the movement of gas may be often distinguished.

*Excessive motory action constitutes Colic or Spasm* (*hyperkinesis*), a condition indicated by symptoms familiar to all professional men, but a condition which should be carefully distinguished from pain as pain. Were I to assert that we can in every instance differentiate between spasm (*cramp*) and pain, I should be asserting an untruth, but for the purposes of this paper it is necessary that I should draw a line between the two states.

*Pain may exist without spasm*--the reverse does not hold good, at least to the ordinary mind. Violent spasmodic contraction of the intestine produces compression of the terminal fibres of the sensory nerves, and a sensation results which may become agonizing and unbearable ; nothwithstanding this, however, the general functions of the body may not be disturbed ; true, the pulse is increased in rapidity, in volume and in strength ; the respiration is hurried, and there is probably more or less perspiration, but these conditions continue only so long as the spasm persists, and are not accompanied by grave systemic disturbance, not even sufficient disturbance to give rise to fever. Undoubtedly, persistent spasm may produce death by exhaustion, and by interfering with the natural functions and with nutrition of the bowels; but of itself, it does not produce death, any more than does the tonic muscular contraction of tetanus. *Pain* is not only exaltation, it is perversion of the normal sensibility of an organ or part, and even in the case of the bowel, frequently exists when it is in a condition of akinesis. On its intensity will depend, to a very large extent, the gravity of cases of colic disorder, as, if it is great and continuous, death may result from nervous exhaustion; and even where recovery takes place, a very marked depressive effect upon the animal is produced, which may remain for some days after the cause of the pain has been removed.

In order that I may better illustrate the symptoms of some of the lesions to which I shall refer, I will here differentiate the manifestation of spasm and pain respectively. Colic spasm is indicated by uneasiness, by constant movement of the hind legs, stamping, kicking, alternate lying and rising, rolling, switching or twisting of the tail, hurried respiration, increased pulse ratio and often perspiration. The animal may look back to the abdomen and, even when lying, throw the head violently against its walls, but it does not, as a rule, show any care for its own well-being by the manner in which it assumes the recumbent posture. Frequently a horse will perambulate his box for a few minutes, go partially down on his knees, rise again and continue his perambulations for a period of several minutes, before he makes up his mind to lie down; but, on the contrary, he will just as frequently dash himself down on the floor of his stall or box, utterly regardless of the consequences, and when down, will throw himself wildly about, fight furiously with his legs and throw his head about as if, for the time, all consciousness was annihilated; he may, too, roll over and assume the dorsal position, but he does not usually preserve it for a longer period than a few seconds, while in the intervals of enteric relaxation he may, to the ordinary observer, present all the appearances characteristic of health.

In colic pain (*enteralgia*), while its manifestations approximate more or less to those of spasm, there is evidence of a greater amount of actual suffering. From the outset greater care is shown in recubation, and when recumbent, the dorsal position, if assumed, is preserved for a period of some minutes; the eye has an anxious expression; the countenance is wistful, and the head is turned carefully to the seat of pain, and laid gently on the part, and this act is often accompanied by a sigh or a moan. It is generally stated that a horse, when suffering from the pain of inflammation, does not seek to produce, but rather to avoid pressure on the abdomen, but this statement does not always hold good; on the contrary, gentle pressure is sometimes sought, and when brought to bear affords a certain degree of relief; if, however, pain continues and increases in intensity, the animal loses all control over itself, dashes violently about, fights desperately, is utterly careless as to the infliction of personal injuries, assumes various abnormal positions, and ultimately becomes delirious. Thus it happens that attempts to judge of the existence or absence of inflammation are rendered futile, and the supposititious statement that a horse suffering from enteritis is extremely careful to avoid concussion or pressure is disproved.

Other manifestations of enteric pain and suffering, are: sitting on the haunches, circum-perambulating, (often for many minutes or even hours), plaiting of the hind legs, backing and pressing the quarters against prominent and fixed objects, groaning, neighing, (often wildly), clenching of the jaws, gnashing of the teeth, muscular tremors and twitchings, frequent attempts at micturition, with straining, sometimes so violent as to produce prolapse of the rectum and rupture of the intestines, or even the abdominal muscles.

Whatever may be the degree or nature of the spasm or pain, every means at our disposal should be had recourse to in order to relieve it, and every care should be taken to prevent the animal injuring either itself or the attendants; and, if possible, it should on no account be allowed to lie down and roll. Exceptions to this rule in dealing with colic affections there are, and they will be alluded to in due course.

It is not necessary to notice in detail the various means at our disposal for the relief of spasm and of pain. I will merely remark that while all ordinary methods should be adopted, we have few more valuable anti-spasmodics and anodines than Chloral Hydrate, Morphia, Aconite and Atropine; and further, that *venesection* and the rectal injection of Amyl Nitrite, Ether, Prussic Acid and Chloroform *are too often neglected.* I have over and over again succeeded in relieving a violent attack of colic by the abstraction of blood, after all the ordinary remedies had failed.

MOTOR DISABILITY—*Akinesis.*—Considering the immense mass of material upon which the colon has, as a rule, to act, it is fortunate that, with the exception of these *temporary* instances associated with indigestion, cases of *colic paralysis* are rare; nevertheless, *permanent* paralysis is occasionally met with. *Temporary paralysis* of the colon always accompanies acute indigestion, (impaction), and is produced by exhaustion of the muscular coat, mainly through the great pressure exerted upon the nerves by ingesta or gas, may also assist in its production. It is with the reservation already noted, indicated by absence of borborygmal sounds and retention of fæces. *Permanent or Chronic paralysis* is, as before observed, rarely met with; or perhaps I should be speaking more accurately if I said, *it is rarely diagnosed* as a condition in itself. My attention was first directed to it about the year '62, by the late Mr. Moore of London. The causes of Chronic Colic Paralysis are, *a*, muscular; *b*, nervous. The muscular coat of the bowel may become the seat of fatty changes or of atrophy as the result of inflammation, or, more probably, impaired nutrition from deficient nerve force. In the vast majority of cases it is produced by feeding, for a long period, upon large quantities of innutritious matter, *e. g.*, poor hay, bean or pea straw and chaff; and the more voracious the animal, the more likely it is to take place. The actual cause of the nervous form of paralysis is more difficult to trace.

No matter how produced, impaired motory function, in however slight degree, is a predisposing cause of colic indigestion, and must be vigorously combated and, if possible, removed.

The means at our disposal for effecting this desirable end are : careful feeding, the occasional administration of laxatives, combined or alternated with nerve stimulants, digestives and carminatives ; moderate but not fatiguing exercise should also be allowed. In acute temporary colic paralysis, a stimulating purge, *e. g.*, Aloes and Calomel may be all that is required to restore the function of the bowel ; in sub-acute and chronic cases, strychnine is, on the whole, the best agent that can be administered; it also may be combined with small doses of aloes and alternated with nitro-hydrochloric acid. Of galvanism I have never made trial. To the use of physostigmata I shall make reference hereafter in speaking of colic indigestion.

### ARREST OF THE SECRETORY AND DIGESTIVE FUNCTION OF THE COLON.

Of the secretory function of this bowel there is little to be said further than that excessive mucous secretion—colic catarrh—is undoubtedly one of the factors in operation in producing diarrhœa ; *vice versa,* arrest of this secretion favors retention of fæces and constipation. That the colon possesses, to a certain extent, digestive functions can scarcely be denied ; were it otherwise, there would scarcely have been the necessity which—judging from the voluminosity of the organ—seems to exist for the prolonged retention of the ingesta within its cavity. Holding, as I do, the opinion that digestion goes on in the colon, I have no hesitation in speaking of arrest of its functions as Colic Indigestion, and in recognizing various degrees of this condition, viz. : Acute, sub-acute and chronic.

### ACUTE COLIC INDIGESTION

May or may not be associated with acute gastric indigestion; the complication materially aggravates the symptoms and adds to the gravity of the case. It is most frequently seen in the heavier classes of horses, and in all animals, defects in the masticatory and insalivatory processes, predispose to the condition as does also, in my opinion, inactivity of the liver, and impairment of nerval force. The great determining cause is the ingestion of larger quantities of food than the gut is capable of dealing with; and especially succulents, green or semi-dried vegetable matter, such as clover, meadow grass, vetches, etc., and above all other materials, perhaps, new hay. Potatoes—especially if diseased or decayed—and boiled foods in inordinate quantity, are frequently the cause of acute colic indigestion.

Following the ingestion in large quantities, of such material as those above enumerated, we have firstly, functional and secondly, motory paralysis followed by decomposition, or fermentation of the ingesta, the liberation of gas, (carbonic acid, carburetted hydrogen and sulphuretted hydrogen) and Tympany.

*Symptoms and Course.*—Acute colic indigestion may be productive of a series of alarming and dangerous symptoms, quickly ending in some fatal lesion, *e. g.*, rupture of the bowel itself or of the diaphragm, or, where gas is evolved, in death by suffocation, or death by exhaustion. The symptoms and conditions presented are aggravated in proportion to the amount of gas given off.

In the early stages, there are symptoms of enteric pain, but not manifested in any particular manner; retention of fæces preceded by relaxative and apparent diarrhœa; inappetence and constant recubation, and, when down, frequently turning the head back to the abdomen; and in some cases rolling on to the back. The animal occasionally gives vent to a grunt, especially if straining is induced by the presence of fæces in the rectum, and small quantities of gas are expelled per anum. The body has a rotund appearance. The animal is dull and its movements are sluggish; the respiration more or less labored. The pulse full, oppressed and increased in frequency. The mucous membranes deepened in color, and the mouth somewhat dry and musty. The temperature is not as a rule materially elevated from 2 to 3° F. at most. There may be frequent attempts to perform micturition.

This condition of things may continue for several hours with intermissions of pain and distress, but in a longer or shorter period the symptoms increase in intensity, the pain becomes more violent, the breathing more labored, and the nostrils widely dilated ; the pulse markedly oppressed and weak, or strong, full and hard ; the body more rotund, and on percussion—especially in the upper right flank—there is marked tympanitic resonance. If relief is not afforded, symptoms of suffocation supervene ; the superficial veins become distended with blood ; the action of the heart irregular, oppressed, and sometimes intermittent ; the mucous membranes very dark in color and the skin bathed in perspiration—it is often cold. The pain at this stage may be agonizing, or only of moderate intensity, and in some cases, there seems to be not only paralysis of the bowel, but even paralysis of the senses and of consciousness, the animal frequently standing with its legs abducted, its head depressed and its ears lopped, while the eye is dull and expressionless.

If the case is associated with gastric tympany, there may be marked eructation with salivation, and the respiratory symptoms are usually aggravated. In all bad cases of tympany, it will be found on rectal exploration being attempted, that it is with difficulty the hand is passed into the bowel, and the distended (tympanitic) Colon can be felt through its walls. Straining is often induced when pushed into the rectum and enemata are seldom retained for a longer period than a few minutes. Death is produced by asphyxia, by collapse, as the result of rupture, and by exhaustion, very exceptionally by inflammation. Metastatic congestion of the lungs or of the feet, may take place and thus afford relief from the bowel symptoms. Where straining is severe, the rectum beeomes prolapsed and somet'mes lacerated or actually ruptured.

TREATMENT OF ACUTE COLIC INDIGESTION.

In treating cases of this type, several important or cardinal principles should be followed, these are: (1), to prevent rolling. (2), to rouse the bowels to action. (3), to relieve the tension in the bowels, and in the circulation. (4), to relieve pain, and guard against inflammation. (5), to prevent sinking, exhaustion.

1. I have no positive objection to any animal lying down when the subject of acute colic, indigestion and tympany, providing it does so quietly, but if recubation is accomplished carelessly and with great force, displacement or rupture will certainly occur, and these contingencies are still more to be dreaded if the animal is allowed to roll over or to assume the dorsal position. To prevent any untoward results from these causes, the patient should be carefully watched and the whip brought into requisition if required.

2. The bowels may be roused into action by the administration of a stimulating cathartic, *e. g.*, aloes (in solution), combined with carminatives and a nerve tonic, as nux vomica. All egesta should be removed, manually, from the rectum and stimulating enemas, as of salt or turpentine, thrown up frequently. Cathartics in reduced doses may be repeated every six hours, and if pain is excessive they should be combined with belladonna, or alternated with opium or morphia. * Friction applied persistently to the abdomen is useful, as is also kneading and the application of some stimulating agent, as ammonia liniment to the skin of the abdomen.

3. Relief of the circulatory and bowel tension, is of paramount importance. If the large venous trunks are surcharged with blood the right heart also becomes overburdened, as do also the pulmonary vessels. The result of this is oppressed heart, pulmonary congestion, imperfect oxidation of blood, and death by syncope or asphyxia; while

* In all severe cases the advisability of having recourse to eserine, may be considered.

great distension of the colon means, in addition, pressure on the diaghragm and indirectly on the large venous trunks, the lungs and the heart. The means at our disposal for the purpose of relieving tension are *a venesection, paracentesis intestinalis.*

Venesection is, in my opinion, too frequently neglected in gastro intestinal disorder. I have already pointed out its value in relieving spasm, and it is none the less valuable in relieving the circulation in the class of cases under consideration, and in favoring the action of our remedial agents, as also in some cases, in combating tympany. The value of venesection was particulary impressed on my mind on one occasion, several years ago, when, having a particularly bad case of gastro-intestinal tympany under my charge, and having dispatched my assistant and the attendants in different directions for such appliances as I deemed necessary for the relief of the case, I was left alone with my patient, or rather I should say, I had one companion and an invariable one, my pocket case, which fortunately contained a fleam. Unaided, I opened the jugular vein, the blood flowed freely and of a very dark color, and in a few minutes the animal was relieved of all the distressing and alarming symptoms. The heart's action became firm, the respiration quiet, eructation ceased, pain was arrested, and gas passed off in large volumes per anum. As an adjurant, I usually administer a full dose of some powerful stimulant—such as ammonia or alcohol—after venesection.

In all severe cases of tympany the operation of *Paracentesis Intestinalis* should be performed, as the relief afforded is immediate and effectual, and serious complications and results are prevented ; not only is pressure at once removed from the diaphragm, and through it from the heart and lungs, but by the removal of the gas from the intestine, the effects of the pressure on the circulation and consequently upon the nutrition of the intestinal walls are avoided. Prolonged distension is always followed by paresis, more or less marked, and thus the period of convalescence is lengthened and a tendency to recurrence or to chronicity is established.

By many practitioners the operation of paracentesis is dreaded and that too because they entertain exaggerated ideas of its gravity and of the danger usually attendant upon wounding such a structure as the peritoneum. The delay which usually takes place in the performance of the operation gives time for serious complications, *e. g.*, pulmonary congestion, enteritis, rupture, displacement, etc., to arise ; and in some cases, nervous and muscular exhaustion is so far advanced as to preclude the possibility of recovery.

The first occasion on which I performed the operation of paracentesis, for remedial purposes, was in the year 1871, in the case of a mule suffering from the effects of an impacted calculus ; several punctures being made in different parts of the abdomen with great temporary benefit. From that time onwards I have frequently performed the operation without, in the great majority of instances, any ill result ; and, (in all cases where it has been performed *sufficiently early* and in which there has been *an absence of complications*) with great benefit. The most annoying result I have met with, and that only on one or two occasions, has been parietal abcess followed by the formation of sinuses.

I have made *post-mortems* of the carcasses of animals in which the operation had been performed at periods varying from several hours to two or three days prior to death, and in only one instance have I seen any evidence of peritonitis having resulted from the operation itself, and in that case there was abundant proof that the health of the animal had been indifferent previously to the fatal attack.

The site of *paracentitis intestinalis* is in one word, at the most salient point or points of distension ; and while the practitioner may adopt this rule as a standard one, he must at the same time remember that in the vast majority of cases the most pronounced distension takes place in the *upper right flank*, and that too for the simple reason that it is the region (right lumbar) of the cæcum caput coli. Before performing the operation, the usual surgical precautions, *i. e.* the removal of the hair, cleansing and disinfection of the skin and the aseptisizing of the trocar and canula should be adopted: a preliminary incision through the skin with the lancet facilitates the introduction of the trocar.

The trocar first employed by me was a bladder (human) trocar, and in preference I use it and recommend it to this day, as it is as nearly as possible the right size; and being curved, the canula is gripped by the bowel, and is less liable to displacement when that organ undergoes collapse. Any small trocar may be used. I have, for instance, frequently used my small combined trocar and canula, milk syphon and exploring needle; as also my aspirator needles. The precise spot in the flank at which the instrument should be introduced is, as before stated, the most prominent part and where the muscle is thinnest. On the introduction of the instrument and the withdrawal of the trocar, a rush of gas usually takes place; occasionally, however, the canula becomes blocked by the contents of the bowel. The liberation of the gas is followed by immediate subsidence of all the more alarming symptoms, and the animal often gives a deep sigh of relief. The gas, as it escapes, may be simply tested by its odor and by the application of the flame of an ignited match for the purpose of determining its chemical composition; evidence of the stage of chemical change in the contents of the bowels being at once afforded by these means. Inasmuch as the sudden liberation of a large volume of gas leads to a material alteration in the physical condition of the circulation and of, respiration whereby syncope or collapse may possibly be produced. I always advise that a tolerably large dose of ammonia carbonate be given in a ball a short time before the operation of paracentesis is performed. The canula may be left *in situ* for a period of from a few minutes to half an hour or an hour; but its prolonged retention is only warranted when the practitioner can himself spare the time to watch it, or has at hand a trustworthy assistant. So long as gas (in puffs) escapes through the canula so long may it be retained with safety, but when, by carefully moving the instrument, its free extremity can be felt rubbing against the outer surface of the bowel it should be at once withdrawn, as it is an easy matter to re-introduce it if necessary.

The introduction of medicament per canula is an operation which should never be resorted to unless there is an absolute certainty that the free end of the canula is still in the cavity of the bowel ; and in no case should the introduction of agents of an irritant character be risked. Ether has been introduced by a French V. S., with good result; it would act admirably as a stimulant, and if it escaped into the peritoneal cavity it would be less irritant than many other agents—but no young practitioner should prejudice his professional status by attempting it—he had better trust to remedies administered in the ordinary way.

The subsequent treatment of the puncture requires little comment ; the application of some antiseptic dressing, once or twice a day, is all that is necessary, under ordinary circumstances; if, however, the tissues around the seat of puncture become inflamed, a cantharides or mylabris blister should be at once applied over an area of several inches, and this should be followed by hot fomentations if the inflammatory action does not subside. Abscesses or sinuses must be treated by ordinary rules.

4. For the relief of pain in cases of acute colic indigestion, the ordinary remedies, as morphia, chloral hydrate, opium, hemp or hyoscyanus, with hot fomentations and the application of anodyne liniments to the abdomen, and rectal injections of chloroform, prussic acid or ether may be employed, and, if inflammation is threatened, aconite and venesection should be brought into requistion.

5. For the purpose of preventing exhaustion, occasional doses of aromatic spirits of ammonia, ether or alcohol may be administered, or ether may be injected into the rectum.

For several days subsequent to an attack of acute colic indigestion, extreme care should be exercised in the general management of the patient, and nerve stimulants and stimulating digestives should be administered. The practitioner, too, should be on the *qui vive* for signs of the advent of sequelæ and combat them vigorously if they arise.

*To be continued.*

