# Correction

**Published:** 1888-01

**Authors:** 


Corrections in Article on "Colic Diseases" in COMPARATIVE JOURNAL for October: p. 356, eight lines from bottom: read relaxation for relaxative.

P. 357, seven and eight lines from bottom : should read straining is often induced when the hand is passed into the rectum.

P. 359, lines four and five should read (a) *venesection* (b) *paracentesis intestinalis;* line seven read : disorders for disorder ; line fourteen from bottom : read adjuvant for adjurant.

P. 361, line eleven, substitute a comma for full stop after *produced.*

P. 362, line three read medicaments for medicament.

